# Who benefits more from an improved social health insurance? Evidence from supplementary high-cost illness insurance in China

**DOI:** 10.1186/s12913-026-15111-y

**Published:** 2026-07-15

**Authors:** Wenhao Fu, Shiju Dong, Zexuan Yu, Jingjie Sun, Peilong Li, Jiajia Li

**Affiliations:** 1 Department of Social Medicine and Health Management, School of Public Health, Cheeloo College of Medicine, Shandong University, Jinan, 250012 China; 2 School of Management, Lanzhou University, Lanzhou, 730000 China; 3 NHC Key Lab of Health Economics and Policy Research (Shandong University), Jinan, 250012 China; 4 Center for Health Management and Policy Research, Shandong University (Shandong Provincial Key New Think Tank), Jinan, 250012 China; 5 Department of Global Health Policy, Graduate School of Medicine, The University of Tokyo, Tokyo, 113-0033 Japan; 6 Medical Management Service Center of Shandong Health Commission, Jinan, China

**Keywords:** Poverty vulnerability, Health insurance, Supplementary insurance, Equity, Difference-in-differences, China

## Abstract

**Background:**

China introduced the Supplementary High-Cost Illness Insurance (SHCII) to strengthen financial protection against medical impoverishment. This study assesses its effectiveness and, critically, examines the distribution of its benefits across population subgroups to identify who benefits more.

**Methods:**

We used 2013 and 2018 survey data from Shandong Province and a difference‑in‑differences design, restricting the sample to hospitalized patients (*n* = 3,136). The treatment group comprised patients whose out‑of‑pocket expenses exceeded city‑specific 2018 SHCII thresholds; the control group comprised those below the thresholds. The outcome was Vulnerability to Expected Poverty (VEP). Subgroup analyses were performed by income, urban‑rural residence, healthcare access, and age.

**Results:**

Patients who benefit from SHCII experienced a significant reduction in VEP relative to those below the threshold (OR = 0.624; average marginal effect = -0.064). The protective effect was statistically significant for low‑income individuals, rural residents, those with healthcare access within 15 min, and adults aged 45 years and above. No significant effect was found for urban residents or for those with poorer geographical access to care.

**Conclusion:**

Patients who benefit from SHCII experienced a greater reduction in poverty vulnerability relative to those below the threshold, with effects concentrated among low‑income, rural, older, and well‑accessed populations. No significant association was observed for urban residents. The findings from Shandong, where policy parameters are broadly consistent with national guidelines, are likely generalizable to other provinces with similar policy designs and rural healthcare infrastructure. Future research is needed to test how different policy parameters affect the results.

**Clinical trial number:**

Not applicable.

## Introduction

Social health insurance (SHI) is a fundamental tool for mitigating the financial risks associated with illness [1–3]. However, a persistent challenge across many systems is the inadequate protection against medical impoverishment, particularly for vulnerable subgroups [4, 5]. For instance, even with extensive coverage, populations in countries like Germany and France still face financial hardship due to high out-of-pocket payments for certain treatments [6–8]. Similarly, in China, despite achieving near-universal basic health insurance coverage [9], high direct medical costs continue to impose significant financial burdens on households facing major illnesses [10].

To address coverage gaps, many systems have developed voluntary supplementary health insurance. However, evidence from countries like Sweden and the UK indicates that such voluntary schemes are often disproportionately purchased by higher-income, healthier, and better-educated groups, seeking faster access or more comfortable care [11, 12]. This can inadvertently exacerbate inequalities in financial protection, as the wealthy augment their coverage while the poor remain exposed to catastrophic costs [6, 13]. In contrast, China’s Supplementary High-Cost Illness Insurance (SHCII), launched in 2015, is a publicly financed scheme integrated into the basic social health insurance for residents (RMI). Its explicit goal is to prevent poverty due to illness, raising a critical and distinct research question: Can such a publicly-managed supplementary scheme provide equitable financial protection, and if so, which population groups benefit the most?

The SHCII was introduced in 2015, a time when despite high basic insurance coverage [14], out-of-pocket health spending remained a leading cause of poverty in China [15]. While evaluations suggest SHCII alleviates financial burden [16], concerns persist regarding the equitable distribution of its benefits. Recent studies indicate that the integration of health insurance schemes can reduce income inequality [17] and that environmental factors may contribute to socioeconomic health disparities [18], yet the distributional effects of China’s SHCII within the health system remain less explored. Some scholars argue that wealthier individuals, with better access to high-quality and higher-cost medical services, might be more likely to trigger SHCII reimbursements, potentially skewing benefits towards them [19]; this concern aligns with the concept of “pro-rich” health inequality observed in other settings [20]. Furthermore, urban-rural disparities in healthcare resources could limit rural residents’ ability to utilize the scheme effectively [21–23].

A key methodological choice in this study is the use of Vulnerability to Expected Poverty (VEP) as the outcome measure. VEP estimates the probability that a household will fall into poverty in the future due to shocks such as illness [24]. Unlike static indicators such as current household income, which captures only a single point in time and cannot anticipate future risks, VEP incorporates household consumption, assets, and exposure to shocks, providing a dynamic assessment of financial vulnerability [25]. Similarly, while catastrophic health expenditure measures whether a household has already incurred high medical costs, it does not reveal the risk of falling into poverty as a consequence [26]. VEP overcomes these limitations by explicitly modeling the ex‑ante probability of impoverishment, making it particularly well‑suited for evaluating policies like SHCII that aim to prevent poverty rather than merely compensate for past expenses [27]. The VEP approach has been extensively validated in Chinese settings, where it has been used to assess the poverty‑reducing effects of health insurance [28], to measure the vulnerability of rural and urban households [29], and to evaluate the impact of relevant policies [30, 31]. Consequently, VEP offers an accurate reflection of financial risk in China’s post‑absolute‑poverty era, where preventing a relapse into poverty has become a central policy priority.

Therefore, this study aims to investigate the distributional benefits of SHCII by addressing two questions: (1) Does SHCII reduce the risk of future poverty? (2) Are its effects heterogeneous across income, urban-rural residence, healthcare accessibility, and age? We employ a quasi-experimental Difference-in-Differences (DID) design using survey data from Shandong Province (2013 and 2018). This study advances the literature in two ways. First, unlike prior work focusing on average effects, we explicitly examine effect subgroup across income, residence, healthcare access, and age. Second, by restricting analysis to hospitalized patients and using city‑specific SHCII thresholds to define high‑cost groups, we adopt a more precise and policy‑relevant design than previous diagnosis‑based approaches. Crucially, we measure impact using Vulnerability to Expected Poverty (VEP), a forward‑looking metric that captures the ex‑ante probability of falling into poverty, which aligns with SHCII’s goal of preventing impoverishment.

## Methods

### Data

This study utilized data from the 2013 and 2018 waves of the China Health Services Survey (CHSS) in Shandong Province. The CHSS employs a multi-stage stratified cluster sampling method and collects comprehensive information on demographics, socioeconomic status, health, and medical insurance [15, 32, 33]. Shandong Province was among the first to implement a province-wide, integrated SHCII, making its data particularly relevant for policy evaluation [34]. Our analysis sample was restricted to individuals enrolled in the Residents’ Basic Medical Insurance (RMI), which is the population eligible for SHCII. After excluding observations with missing key variables, the full sample contained 39,006 individuals. For the main analysis, we further restricted the sample to hospitalized patients (*n* = 3,136). According to Shandong’s SHCII policy, the reimbursement threshold is calculated based on annual cumulative out‑of‑pocket expenses from both inpatient care and qualified outpatient chronic disease services. Ideally, the treatment group should be identified using cumulative expenses from both sources. However, in the CHSS data, annual cumulative outpatient expenses over a 12‑month period are subject to recall error and cannot be reliably aggregated. To ensure accurate classification of the treatment group (high‑cost vs. low‑cost) and to avoid misclassification bias, we therefore restricted the analysis to hospitalized patients. Additionally, focusing on this group ensures that both the treatment and control arms consist of individuals who have experienced a condition serious enough to require inpatient care, which further improves comparability.

### Vulnerability as expected poverty

The concept of “VEP” was first introduced by the World Bank in 2002. It is defined as “the probability that an individual or a household suffers a loss of wealth or a decline in quality of life below a socially acceptable level due to certain risks” [35]. In 2018, Gallardo et al. conducted a comprehensive analysis of VEP prediction methods and established identification criteria [36]. According to the research of Gallardo and Ouadika [36, 37], we employed VEP to measure the likelihood of an individual falling into poverty in the future. Using the estimation method proposed by Chaudhuri et al. [38], we calculated the probability of individual $\:h{\prime\:}\mathrm{s}$ VEP at the time $\:\boldsymbol{t}\:+\:\boldsymbol{j}$. The basic formula for measuring VEP is as follows.

1 $$\:{\boldsymbol{V}}_{\boldsymbol{h}\boldsymbol{t}}={\boldsymbol{P}}_{\boldsymbol{r}}(\mathbf{ln}{\boldsymbol{Y}}_{\boldsymbol{h},\boldsymbol{t}+\boldsymbol{j}}<\mathbf{ln}\boldsymbol{z})$$

$\:{\boldsymbol{V}}_{\boldsymbol{h}\boldsymbol{t}}$ represents the vulnerability of individual * h* in period * t*, and refers to the probability that the individual’s future income ***(***$\:{\boldsymbol{Y}}_{\boldsymbol{h},\boldsymbol{t}+\boldsymbol{j}}$**)** falls below the threshold value *z*. Future income could be expressed as a function of the observable variable $\:{X}_{h}$ and the error term $\:{\epsilon\:}_{h}$ of its impact factors, representing its impact factors. By combining this with the basic formula (1) for measuring VEP, formula (2) can be derived.

2 $$\:{\boldsymbol{V}}_{\boldsymbol{h}\boldsymbol{t}}={\boldsymbol{P}}_{\boldsymbol{r}}\left({\boldsymbol{Y}}_{\boldsymbol{h},\boldsymbol{t}+\boldsymbol{j}}=\boldsymbol{f}\left({\boldsymbol{X}}_{\boldsymbol{h}},{\boldsymbol{\beta\:}}_{\boldsymbol{t}},{\boldsymbol{\epsilon\:}}_{\boldsymbol{h}}\right)<\mathbf{l}\mathbf{n}\boldsymbol{z}\right)$$

$\:{\boldsymbol{X}}_{\boldsymbol{h}}$ represents the vector of observable variables, $\:{\boldsymbol{\beta\:}}_{\boldsymbol{t}}$ denotes the parameter vector of observable economic variables, and $\:{\epsilon\:}_{h}$ is the error term capturing the shock factor. To estimate the income equation, we employed the three-stage feasible generalized least squares (FGLS) method to measure VEP. In the first step, we estimated the regression by taking the logarithm of the per capita annual household income, as shown in formula (3):

3 $$\:\boldsymbol{ln}{\boldsymbol{Y}}_{\boldsymbol{h}\boldsymbol{t}}={\boldsymbol{X}}_{\boldsymbol{h}}\boldsymbol{\beta\:}+{\boldsymbol{\epsilon\:}}_{\boldsymbol{h}}$$

where $\:{\boldsymbol{Y}}_{\boldsymbol{h}\boldsymbol{t}}$ represents the individual’s income in period ***t***, and $\:{\boldsymbol{X}}_{\boldsymbol{h}}$ denotes a vector of individual and household characteristics. According to previous studies, vulnerability factors related to health are categorized into three dimensions: household coping capacity, health shocks, and health support systems [39, 40]. In this study, household coping capacity includes factors such as income, expenditure, gender, age, education level, marital status, occupation, and ethnic group. Health shocks are represented by variables such as the EQ-5D score, body mass index, smoking status, alcohol consumption, exercising, and dental hygiene. The community health support system comprises the type of drinking water, type of toilet, distance to the nearest health facility, availability of health records, type of household registration, and whether the individual resides in their hometown. The definitions and types of variables used to predict VEP are presented in Table 1. Subsequently, we performed regression estimation by substituting the squared residuals from Eq. (3) into Eq. (4), we obtained estimates of the predictor variable $\:{\boldsymbol{Y}}_{\boldsymbol{h}}$ and the residual term $\:\boldsymbol{\sigma\:}$.

4 $$\:{\widehat{\boldsymbol{\sigma\:}}}_{\boldsymbol{o}\boldsymbol{l}\boldsymbol{s},\boldsymbol{h}}^{2}={\boldsymbol{X}}_{\boldsymbol{h}}\widehat{\boldsymbol{\theta\:}}+{\widehat{\boldsymbol{\eta\:}}}_{\boldsymbol{h}}$$

In the second step, based on formula (3) and (4), we applied the logarithm of income and the square of residual for FGLS estimation to obtain the coefficients of estimated variables $\:\left({\widehat{\boldsymbol{\beta\:}}}_{\boldsymbol{F}\boldsymbol{G}\boldsymbol{L}\boldsymbol{S}}\right)$ and $\:\left({\widehat{\boldsymbol{\theta\:}}}_{\boldsymbol{F}\boldsymbol{G}\boldsymbol{L}\boldsymbol{S}}\right)$. These coefficients were then substituted into formulas (5) and (6) to calculate the logarithmic expected value of income $\:\boldsymbol{E}\left(\left.\mathbf{ln}{\widehat{\boldsymbol{Y}}}_{\boldsymbol{h}}\right|{\boldsymbol{X}}_{\boldsymbol{h}}\right)$ and variance $\:\boldsymbol{Var}\left[\left(\boldsymbol{ln}{\widehat{\boldsymbol{Y}}}_{\boldsymbol{h}}|{\boldsymbol{X}}_{\boldsymbol{h}}\right)\right]$.

5 $$\:\boldsymbol{E}\left(\left.\boldsymbol{ln}{\widehat{\boldsymbol{Y}}}_{\boldsymbol{h}}\right|{\boldsymbol{X}}_{\boldsymbol{h}}\right)={\boldsymbol{X}}_{\boldsymbol{h}}{\widehat{\boldsymbol{\beta\:}}}_{\boldsymbol{F}\boldsymbol{G}\boldsymbol{L}\boldsymbol{S}}$$

6 $$\:\boldsymbol{Var}\left[\left(\boldsymbol{ln}{\widehat{\boldsymbol{Y}}}_{\boldsymbol{h}}|{\boldsymbol{X}}_{\boldsymbol{h}}\right)\right]={\boldsymbol{\sigma\:}}_{\boldsymbol{h}}^{2}={\boldsymbol{X}}_{\boldsymbol{h}}{\widehat{\boldsymbol{\theta\:}}}_{\boldsymbol{F}\boldsymbol{G}\boldsymbol{L}\boldsymbol{S}}$$

In the third step, we assumed that the logarithm of income was normally distributed and calculated the VEP under poverty line according to formula (7).

7 $$\:{\widehat{\boldsymbol{V}}}_{\boldsymbol{h}}={\widehat{\boldsymbol{P}}}_{\boldsymbol{r}}\left(\boldsymbol{ln}{\boldsymbol{Y}}_{\boldsymbol{h}}<\boldsymbol{ln}\boldsymbol{z}\right)=\boldsymbol{\varPhi\:}\left(\frac{\boldsymbol{ln}\boldsymbol{z}-{\boldsymbol{X}}_{\boldsymbol{h}}{\widehat{\boldsymbol{\beta\:}}}_{\boldsymbol{F}\boldsymbol{G}\boldsymbol{L}\boldsymbol{S}}}{\sqrt{{\boldsymbol{X}}_{\boldsymbol{h}}{\widehat{\boldsymbol{\theta\:}}}_{\boldsymbol{F}\boldsymbol{G}\boldsymbol{L}\boldsymbol{S}}}}\right)$$

For the estimation, we used the minimum income standards of the year as the poverty line: $863.52 for the urban population and $639.14 for the rural population in the 2013 Health Services Survey [41]; similarly, $968.36 for the urban population and $636.35 for the rural population in the 2018 Health Services Survey [42]. In this study, we defined the highly vulnerable group as individuals whose predicted future annual per capita household income had a logarithm of the probability of being below the poverty line by more than 50%, classifying them as VEP.

**Table 1:** Variable definition and type of predicted VEP

	Variable	Variable definition and type
Household coping capacity	Income	The logarithm of per capita annual household income
	Expenditure	The logarithm of per capita annual household expenditure
	Marital status	0 = other, 1 = married
	Occupation	0 = No (Student, retired or unemployed), 1 = Yes
	Type of ethnic group	0 = Other, 1 = Ethnic Han
Health shocks	Body Mass Index	1 = Thin (18.5), 2 = Normal (18.5 ≤ BMI < 24.9), 3 = Overweight (25.0 ≤ BMI < 29.9), 4 = Obese (BMI ≥ 30.0)
	Smoking	0 = No, 1 = Yes
	Drinking	0 = No, 1 = Yes
	Exercising	0 = No, 1 = Yes
	Brushing teeth	0 = No, 1 = Yes
Health support systems	Type of drinking water	0 = other, 1 = tap-water
	Type of toilet	0 = other, 1 = Dry toilet, 2 = Flushable toilet
	Nearest health facility distance	1 = less than 1 km, 2 = 1–2 km, 3 = 2–3 km, 4 = 4–5 km, 5 = more than 5 km
	Availability of health records	0 = No, 1 = Yes
	Type of household registration	0 = Other, 1 = Rural, 2 = Urban
	Live in the hometown	0 = No, 1 = Yes

### Study design and group definition

The SHCII reimbursement threshold varied across cities in Shandong Province in 2018. According to official documents from the Shandong Provincial Government and the relevant municipal policy departments, the thresholds were as follows: 14,000 RMB (≈ 2,000 USD) in Jinan; 18,000 RMB (≈ 2,570 USD) in Qingdao, Zibo and Weihai; 16,000 RMB (≈ 2,290 USD) in Dongying and Yantai; 15,000 RMB (≈ 2,140 USD) in Dezhou; 12,000 RMB (≈ 1,710 USD) in Zaozhuang, Weifang, Jining, Rizhao, Linyi, Liaocheng, Binzhou and Heze; and 10,000 RMB (≈ 1,430 USD) in Tai’an.

Out‑of‑pocket medical expenses were calculated as total inpatient cost minus basic medical insurance reimbursement. All cost and reimbursement data refer to the 12 months preceding the survey interview. A hospitalized patient was assigned to the treatment (high‑cost) group if their out‑of‑pocket expenses exceeded the city‑specific 2018 SHCII threshold; otherwise, they were assigned to the control (low‑cost) group. This classification was applied identically to both the 2013 and 2018 samples. Thus, in 2013 the treatment group consisted of patients whose expenses, when adjusted to 2018 prices, met or exceeded the city‑specific SHCII thresholds. This fixed grouping is standard in Difference‑in‑Differences (DID) designs and allows the interaction term to capture the differential change in outcomes over time between the two groups.

### Variables and statistical model

The primary outcome is the binary Vulnerability to Expected Poverty (VEP) indicator. To estimate the policy’s association with VEP, we defined a binary treatment variable high-cost (1 for the high‑cost group and 0 for the low‑cost group) and a binary time indicator post (1 for 2018 and 0 for 2013). The coefficient of primary interest is the interaction term high‑cost multiplied by post, which we denote as the DID estimator. The analysis also adjusted for a set of covariates measured at the time of the survey, including age, sex, education level, marital status, whether a healthcare facility is reachable within 15 min, enrollment in commercial health insurance, presence of a chronic disease, the EQ‑5D health utility index, income group, and urban/rural residence. To avoid multicollinearity, variables that had been used to construct the VEP index were deliberately excluded from the covariate set, because the VEP index already incorporates them.

To estimate the effect of SHCII, we employ a DID framework [43, 44]. A logistic regression model with a group‑by‑time interaction term was estimated. The econometric model is specified as follows:

9 $$\begin{gathered} {Y_{it}} = \alpha + \delta \cdot high\_cos{t_i} \hfill \\ \times Pos{t_t} + \lambda \cdot Intensit{y_i} \hfill \\ + \mu \cdot p{\boldsymbol{os}}{{\boldsymbol{t}}_t} + \eta '{X_{it}} + {\varepsilon _{it}} \hfill \\ \end{gathered} $$

where $\:\boldsymbol{h}\boldsymbol{i}\boldsymbol{g}\boldsymbol{h}\_\boldsymbol{c}\boldsymbol{o}\boldsymbol{s}\boldsymbol{t}$ equals 1 for the high‑cost group and 0 for the low‑cost group, $\:\boldsymbol{p}\boldsymbol{o}\boldsymbol{s}\boldsymbol{t}$ equals 1 for 2018 and 0 for 2013, and the interaction term $\:\boldsymbol{h}\boldsymbol{i}\boldsymbol{g}\boldsymbol{h}\_\boldsymbol{c}\boldsymbol{o}\boldsymbol{s}\boldsymbol{t}\:\times\:\:\boldsymbol{p}\boldsymbol{o}\boldsymbol{s}\boldsymbol{t}$ (denoted as DID) captures the group‑specific temporal difference in the odds of VEP. $\:{\boldsymbol{X}}_{\boldsymbol{i}\boldsymbol{t}}$ represents other control variables affecting VEP, while $\:{\boldsymbol{\epsilon\:}}_{\boldsymbol{i}\boldsymbol{t}}$ denotes random error term.

To facilitate policy interpretation of the magnitude of the association, we computed two additional effect sizes. First, the average marginal effect (AME) of the interaction term was calculated, which gives the average change in the predicted probability of VEP associated with the interaction term. Second, the average treatment effect on the treated (ATT) was estimated using propensity score matching; this measure provides the average change in the probability of VEP specifically for the high‑cost group.

### Subgroup and sensitivity analyses

To examine whether the association between SHCII and VEP varies across key population characteristics, we estimated the same logistic model separately for subgroups defined by income level, residence, healthcare accessibility, and age. These subgroups were selected based on prior literature and policy relevance: income level captures the pro‑poor targeting of SHCII [45]; residence (rural vs. urban) reflects persistent disparities in healthcare resources and financial protection [46]; healthcare accessibility (whether a facility is reachable within 15 min) indicates the role of geographic barriers in moderating insurance effectiveness [23, 47, 48]; and age (45 years as the cut‑off) corresponds to the elevated health risks and medical needs associated with middle and older adulthood [49, 50]. For each subgroup, the DID estimator was re‑estimated using the same covariate set as in the primary model.

Several sensitivity analyses were conducted. First, to verify that the DID estimates are not driven by a particular matching technique, we implemented the PSM‑DID estimator using three alternative matching algorithms: 1:1 nearest‑neighbor matching, radius matching, and kernel matching. The results across these algorithms were compared to assess consistency. Second, we performed a permutation‑based placebo test to examine whether the observed association could be due to chance. In this test, the treatment indicator was randomly reassigned 500 times while preserving the covariate structure. Because logistic models frequently failed to converge under random assignment, a linear probability model was used for this test. The distribution of the 500 placebo coefficients was compared to the true coefficient obtained from the original model. All analyses were performed using Stata 14.0.

## Results

### Characteristics of sample

Table 2 presents the descriptive statistics of the study sample by group (high‑cost vs. low‑cost) and year (2013 vs. 2018). In 2013, the high‑cost group (*n* = 200) had a mean VEP of 0.175, while the low‑cost group (*n* = 1,282) had a mean VEP of 0.214. In 2018, the mean VEP increased to 0.205 in the high‑cost group (*n* = 298) and to 0.296 in the low‑cost group (*n* = 1,356). Thus, both groups experienced an increase in vulnerability over time, but the increase was smaller in the high‑cost group (0.030) than in the low‑cost group (0.082), suggesting a potential protective pattern associated with SHCII.

The two groups were broadly comparable across most baseline characteristics. The high‑cost group was slightly older (mean age 58.36 vs. 56.91 in 2013) and had a higher proportion of chronic disease (0.625 vs. 0.555). The proportion of female was lower in the high‑cost group (0.505 vs. 0.606). Education, marital status, access to care within 15 min, commercial insurance, EQ‑5D score [51], income group, and urban/rural residence showed only minor differences. The distributions were similar in 2018.

Despite the general upward trend in healthcare costs, the out‑of‑pocket expenses exhibited different patterns between the two groups. From 2013 to 2018, the mean out‑of‑pocket expense in the high‑cost group decreased from 38,085.60 RMB to 37,291.78 RMB, while that in the low‑cost group increased from 4,491.32 RMB to 5,541.27 RMB.

**Table 2:** Descriptive statistics of hospitalized patients by high‑cost and low‑cost groups

Variable	Definition	Pre-policy		Post-policy	
		High-cost	Low-cost	High-cost	Low-cost
VEP		0.175	0.214	0.205	0.296
Gender	1 = female	0.505	0.606	0.507	0.633
Age	years	58.36	56.906	59.91	57.03
Education level					
Never attend any school	1 = Never attend any school	0.295	0.284	0.205	0.261
Primary school	1 = Primary school	0.270	0.308	0.352	0.262
Junior middle school	1 = Junior middle school	0.295	0.306	0.319	0.339
Senior middle school and technical school	1 = Senior middle school and technical school	0.130	0.090	0.107	0.111
Junior college or above	1 = Junior college or above	0.010	0.012	0.017	0.027
Marriage	1 = married	0.840	0.844	0.903	0.876
Medical resources available	1 = Within 15 min	0.835	0.874	0.913	0.904
Commercial health insurance	1 = Yes (Have commercial health insurance)	0.075	0.069	0.111	0.105
Chronic disease	1 = Yes (Have a chronic disease)	0.625	0.555	0.668	0.578
EQ-5D	EQ-5D score	0.870	0.932	0.904	0.927
Income group	1 = Middle income and above	0.310	0.246	0.312	0.305
Rural or urban	1 = Urban	0.430	0.429	0.473	0.403
Out of pocket	RMB	38085.60	4491.32	37291.78	5541.27
N		200	1282	298	1356

**Fig. 1:**
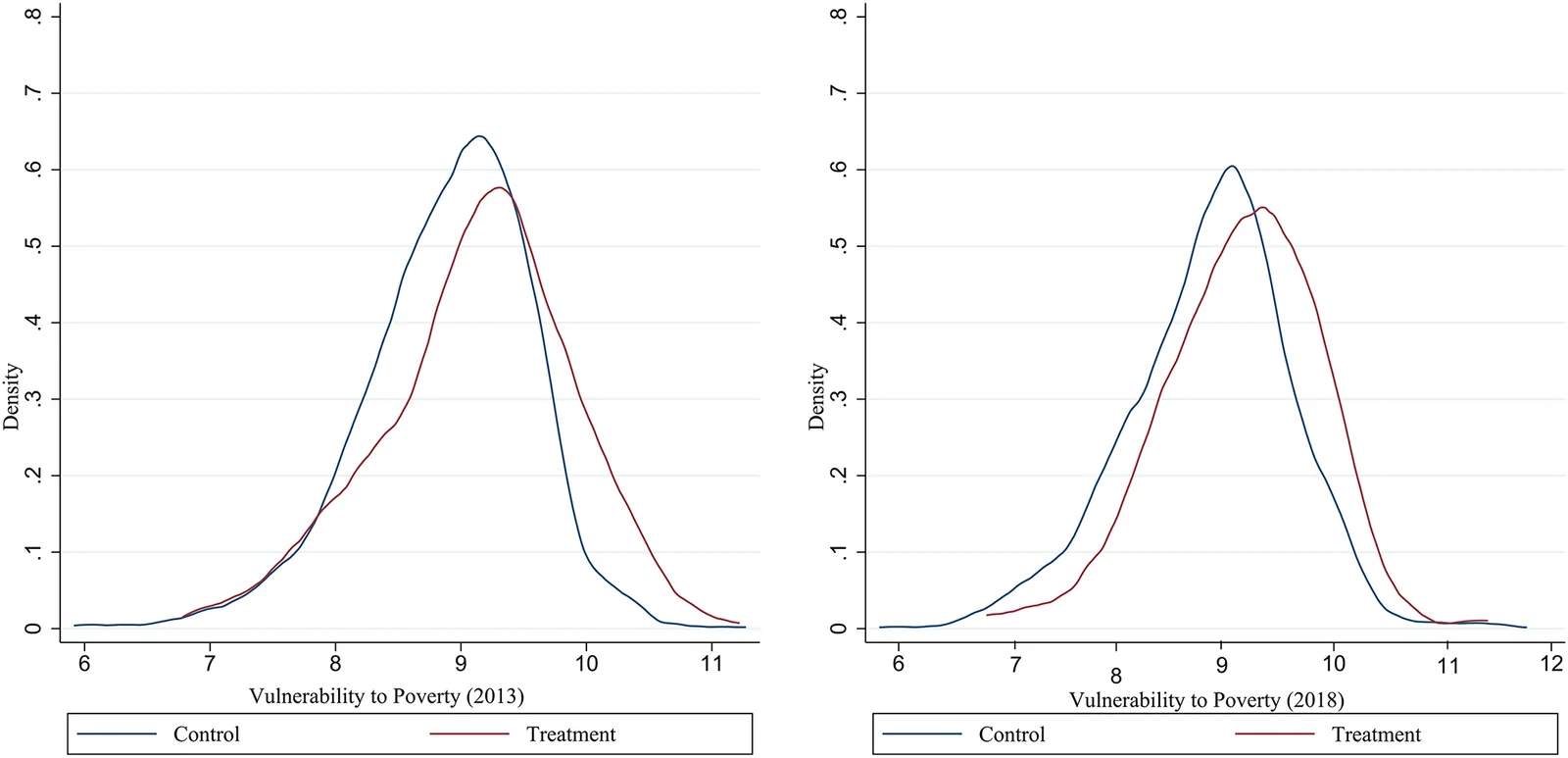
Kernel density estimates of VEP

Figure 1 presents kernel density estimates of VEP for the high‑cost and low‑cost groups in 2013 and 2018. In 2013 (left panel), the distribution for the low‑cost group (blue) is shifted slightly leftward relative to the high‑cost group (red), indicating lower baseline vulnerability. By 2018 (right panel), the low‑cost group’s distribution becomes markedly more dispersed, with a lower peak and a rightward shift, reflecting a larger increase in vulnerability. In contrast, the high‑cost group’s distribution shows only a modest rightward shift and a slightly thicker left tail, consistent with a smaller increase in vulnerability. These patterns align with the mean VEP changes reported in Table 2 and support the validity of the DID approach used to estimate the association between SHCII and VEP.

### Regression results of DID

The interaction term high-cost × 2018 (DID) captures the differential change in VEP from 2013 to 2018 between the high‑cost and low‑cost groups. It measures whether the temporal change in VEP for patients who exceeded the SHCII threshold is significantly different from that for patients who remained below the threshold. Table 3 presents the logistic regression results for the association between the DID and VEP. This estimate (OR = 0.624) reflects the differential change in VEP over time between the high‑cost and low‑cost groups, and should not be interpreted as suggesting that higher medical expenses directly reduce vulnerability.

Among the covariates, older age was associated with higher VEP (OR = 1.041, *p* < 0.001). Female sex was a protective factor (OR = 0.722, *p* < 0.001). Compared with never having attended any school, higher education levels were generally protective: junior middle school (OR = 0.768, *p* < 0.10), senior middle school (OR = 0.642, *p* < 0.10). However, junior college or above showed an unexpected positive association (OR = 2.126, *p* < 0.10). Marital status, access to care within 15 min, commercial health insurance, and chronic disease were not statistically significant. A higher EQ‑5D score was strongly protective (OR = 0.018, *p* < 0.001). Being in the middle‑ or high‑income group (OR = 0.055, *p* < 0.001) and urban residence (OR = 1.841, *p* < 0.001) were significantly associated with VEP, with urban residents having higher vulnerability.

**Table 3:** Logistic regression results for the association between the DID and VEP

Variable	OR(SE)
DID	0.624** (0.115)
Gender: Female	0.722*** (0.079)
Age	1.041*** (0.005)
Education level: Never attend any school	
Never attend any school	0.675*** (0.083)
Junior middle school	0.768* (0.116)
Senior middle school	0.642* (0.150)
Junior college or above	2.126* (0.941)
Marriage: Married	0.813 (0.114)
Medical resources available: Within 15 min	0.832 (0.114)
Commercial health insurance: Yes	0.802 (0.177)
Chronic disease	1.043 (0.114)
EQ-5D	0.018*** (0.007)
Income group: Middle income and above	0.055*** (0.013)
Rural or urban: Urban	1.841*** (0.181)
Constants	2.698 (1.663)
N	3136

∗∗∗ *p* < 0.01, ∗∗ *p* < 0.05, ∗ *p* < 0.1

Table 4 reports the average marginal effect (AME) and the average treatment effect on the treated (ATT) of the DID on the probability of VEP. The AME was − 0.064 (*p* < 0.05), indicating that, on average across the full sample, the high‑cost group experienced a 6.4% point reduction in the predicted probability of VEP compared to the low‑cost group. The ATT was − 0.078 (*p* < 0.05), meaning that among the treated (high‑cost) individuals, the policy was associated with a 7.8% point reduction in VEP probability. Both effect sizes were statistically significant and complement the odds ratio reported in Table 4 by quantifying the absolute risk reduction on a probability scale.

**Table 4:** Average marginal effect (AME) and average treatment effect on the treated (ATT) of DID on VEP

Variable	AME(SE)	ATT(SE)
DID	-0.064** (0.025)	-0.078** (0.037)
Control variable	yes	yes
N	3136	3136

Notes: AME computed using margins, dy/dx(DID); ATT computed using teffects psmatch, atet. Both adjusted for same covariates as Table 3. ∗∗∗ *p* < 0.01, ∗∗ *p* < 0.05, ∗ *p* < 0.1

### Subgroup analysis of the impact of SHCII on VEP

**Fig. 2:**
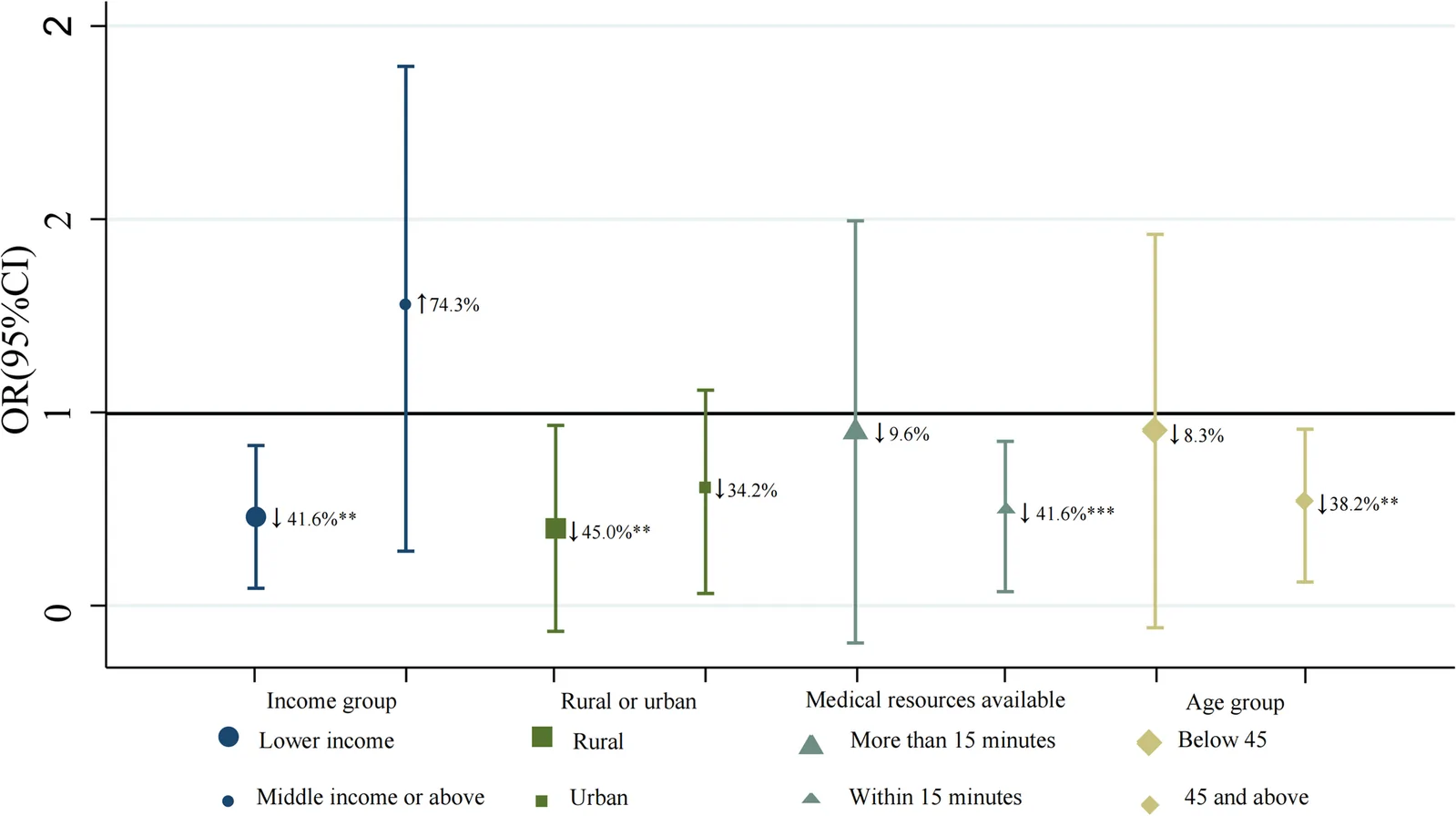
Subgroup analysis of DID effects

Table 5 and Fig. 2 present subgroup analyses of the DID stratified by income level, residence, healthcare accessibility, and age. For income level (Panel A), the DID odds ratio was 0.584 (*p* < 0.05) for the low‑income group, corresponding to a 41.6% reduction in the odds of VEP. For the middle‑ and high‑income group, the OR was 1.743 (*p* > 0.10), which was not statistically significant. For residence (Panel B), rural residents showed a significant protective effect (OR = 0.550, *p* < 0.05), while urban residents did not (OR = 0.658, *p* > 0.10). For healthcare accessibility (Panel C), among those with access to care within 15 min, the DID OR was 0.584 (*p* < 0.01), indicating a 41.6% reduction. Among those living more than 15 min away, the OR was 0.904 (*p* > 0.10), not significant. For age group (Panel D), individuals aged 45 and above had a significant OR of 0.618 (*p* < 0.05), a 38.2% reduction, whereas those below 45 showed no significant effect (OR = 0.917, *p* > 0.10).

These findings (Fig. 2) demonstrate that the protective association of SHCII is concentrated among low‑income, rural, good‑access, and older populations, with no statistically significant effects observed for their counterparts.

**Table 5:** Subgroup analysis of the DID by income, residence, healthcare access, and age

Variable	DID	Control variable	*N*
Panel A: Income group			
(1) Lower income	0.584** (0.110)	Yes	2252
(2) Middle income or above	1.743 (1.081)	Yes	853
Panel B: Rural or urban			
(3) Rural	0.550** (0.150)	Yes	1790
(4) Urban	0.658 (0.177)	Yes	1323
Panel C: Medical resources available			
(5) More than 15 min	0.904 (0.504)	Yes	347
(6) Within 15 min	0.584*** (0.116)	Yes	2786
Panel D: Age group			
(7) Below 45	0.917 (0.472)	Yes	444
(8) 45 and above	0.618** (0.124)	Yes	2443

∗∗∗ *p* < 0.01, ∗∗ *p* < 0.05, ∗ *p* < 0.1

### Robustness tests

Table 6 presents the results of sensitivity analyses using three alternative propensity score matching algorithms (1:1 nearest‑neighbor, radius, and kernel matching). Across all three methods, the DID odds ratio remained stable at 0.625 (*p* < 0.05), consistent with the primary estimate reported in Table 3. The sample sizes were 3,134 for nearest‑neighbor and kernel matching, and 3,130 for radius matching. This consistency indicates that the main finding is not sensitive to the choice of matching technique.

**Table 6:** Robustness checks of DID using different propensity score matching algorithm

Variable	One-to-one matching	Radius matching	kernel matching
DID	0.625** (0.115)	0.625** (0.115)	0.625** (0.115)
Control variable	Yes	Yes	Yes
N	3134	3130	3134

∗∗∗ *p* < 0.01, ∗∗ *p* < 0.05, ∗ *p* < 0.1

**Fig. 3:**
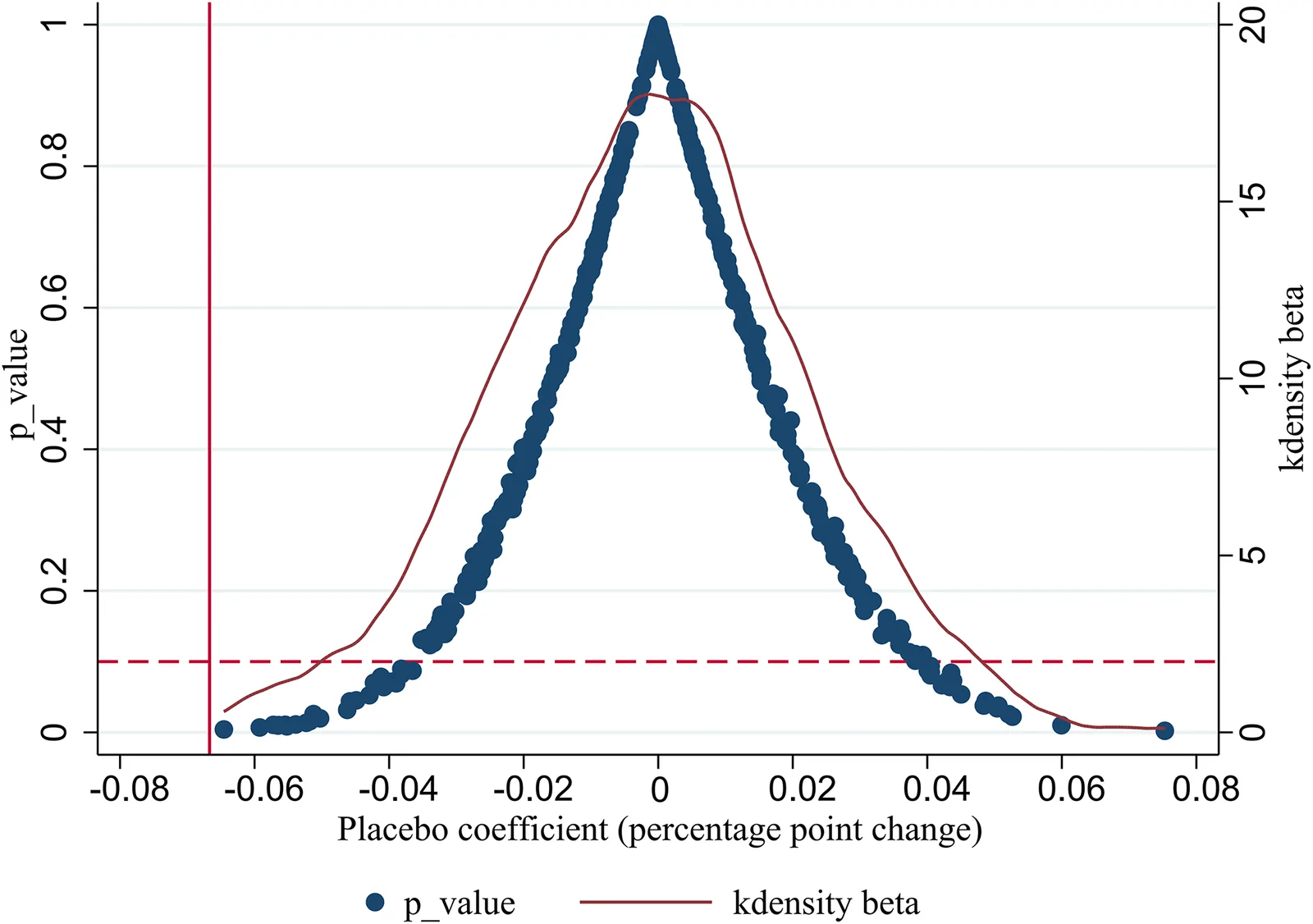
Placebo test distribution of simulated coefficients

To assess whether the estimated association between SHCII and VEP could be driven by chance, we conducted a permutation based placebo test. Figure 3 displays the kernel density of the 500 placebo coefficients (gray curve), along with the true coefficient from our main analysis (red vertical line, -0.067). The distribution of placebo coefficients is centered around zero. This strong evidence indicates that the observed association between SHCII and reduced poverty vulnerability is extremely unlikely to be driven by chance or by unobserved confounding, supporting the robustness of our main finding.

To further assess whether the main finding is sensitive to sample composition, we re‑estimated the DID model in two subsamples defined by out‑of‑pocket expense ranges that cover the majority of the respective cost groups. The first subsample included patients with expenses above the lower quartile (2,884 RMB, *n* = 2,351), excluding the lowest 25% of observations to focus on patients with non‑trivial hospitalization costs. The second subsample included patients with expenses below the 90th percentile (20,000 RMB, *n* = 2,773), covering the vast majority of low‑cost patients while excluding extreme high‑expenditure outliers. As shown in Table 7, the DID interaction term remained statistically significant in both subsamples, with odds ratios of 0.662 (*p* < 0.05) and 0.583 (*p* < 0.10), respectively. These estimates are consistent with the primary estimate (OR = 0.624). The results indicate that the protective association is robust across different sample compositions and is not driven by a particular out‑of‑pocket expense range.

**Table 7:** Robustness checks of DID estimates by out‑of‑pocket expense ranges

Variable	Expenses > 2884 RMB	Expenses < 20,000 RMB
DID	0.662** (0.122)	0.583* (0.185)
Control variable	Yes	Yes
N	2351	2773

∗∗∗ *p* < 0.01, ∗∗ *p* < 0.05, ∗ *p* < 0.1

## Discussion

Our subgroup analyses revealed substantial subgroup in the association between SHCII and VEP. The protective effect was statistically significant for low‑income individuals, rural residents, those with healthcare access within 15 min, and adults aged 45 and above. In contrast, no significant effect was found for middle‑/high‑income individuals, urban residents, those with poorer access, or younger adults. These patterns suggest that SHCII’s benefits are not uniform and are shaped by pre‑existing socioeconomic and geographical disparities.

The pronounced effect of SHCII among low-income groups is a noteworthy success of its policy design. Unlike voluntary supplementary insurance schemes in many high-income countries, which often exacerbate inequality by catering to the wealthy, SHCII’s publicly-funded, automatic enrollment model appears to have achieved pro-poor targeting. This aligns with its foundational objective of preventing a “medical poverty trap” [52–54]. The mechanism likely involves the policy’s high reimbursement rate after a high deductible, which effectively cushions low-income households against catastrophic expenditures that would otherwise deplete their limited financial reserves [55, 56], thereby preserving their capacity for future income generation [16]. This stands in sharp contrast to the common use of supplemental insurance by affluent groups for enhanced comfort or convenience.

Contrary to our initial expectation, the DID estimate was statistically significant for rural residents but not for urban residents. This pattern may reflect differences in baseline financial protection: urban residents typically have more diversified income sources, leaving less room for SHCII to further reduce vulnerability [57, 58]. For rural residents, who often face lower reimbursement, fewer safety nets, and higher exposure to catastrophic costs, SHCII provides a meaningful reduction in poverty vulnerability [59]. However, even with a significant effect, structural barriers such as limited reimbursement for informal care, high indirect costs (transportation, accommodation, lost workdays), and weak primary care capacity still constrain the policy’s full potential [60]. Therefore, while SHCII is effective, achieving equitable protection in rural areas requires complementary investments in healthcare infrastructure, benefit design expansion, and measures to reduce non-medical expenses [61].

The stronger treatment effect observed among those living within 15 min of a health facility requires a nuanced interpretation. Proximity likely does not simply enable cheaper, earlier treatment for the specific illnesses covered by SHCII (e.g., advanced cancer). Instead, it may facilitate higher overall healthcare utilization, including follow-up visits, management of comorbidities, and preventive services [62], all of which can generate costs that become eligible for SHCII reimbursement. Furthermore, easy access supports continuity of care, allowing for better chronic disease management and potentially avoiding some catastrophic acute episodes [63]. Thus, geographic accessibility may act as a multiplier for insurance effectiveness by enabling individuals to fully engage with the health system. Conversely, for those with poor access, the financial and time costs of reaching care may deter necessary service use, rendering the insurance card effectively inert [64], a phenomenon known as “underutilization despite coverage.”

The significant effect for individuals aged 45 and above underscores the policy’s relevance in the context of population aging and the associated rise in chronic disease burden [65]. This group faces elevated health risks and medical needs, making them prime targets for financial protection. Our findings also reinforce that vulnerability is multifaceted [66]. Factors such as lower education, poor health (low EQ-5D score), and lack of commercial supplementary insurance were independently associated with higher VEP [50]. This highlights that medical impoverishment risk arises from a confluence of socioeconomic, health, and access-related disadvantages, suggesting the need for layered, multi-sectoral poverty prevention strategies beyond health insurance alone [67, 68].

Our findings have three main policy implications. First, the significant protective effects for low income, rural, older, and well accessed populations indicate that SHCII successfully reaches many of its intended beneficiaries. To build on this success, policymakers should consider tailoring the benefit package to further reduce out of pocket costs for these groups, for example by lowering effective deductibles for low-income rural households or expanding the reimbursement catalogue to cover essential outpatient drugs and primary care services [21]. Second, strengthening primary care capacity within integrated service networks is crucial to bring quality, reimbursable care closer to rural populations and to manage illnesses proactively before they become catastrophic [69, 70]. Third, the lack of a statistically significant effect for urban residents does not imply that SHCII is unnecessary in cities. For urban settings, the policy focus could shift to improving the efficiency of claims processing, enhancing financial literacy about supplementary insurance, and addressing any remaining gaps for vulnerable urban subgroups such as migrant workers or the near poor [71]. Overall, SHCII is a valuable tool, but its effectiveness across provinces will depend on specific policy parameters (e.g., deductibles, co‑payment rates) and the strength of rural primary care infrastructure. Policy makers in regions considering similar schemes should carefully calibrate these parameters to local conditions. Our results suggest that provinces with lower deductibles or more generous co‑payment rates may observe stronger protective effects, while those with higher deductibles might see weaker effects.

### Limitations

This study has several limitations. First, the repeated cross‑sectional data with only two time points prevent us from tracking individual‑level changes in VEP or formally testing the parallel trends assumption, and unobserved confounding cannot be completely ruled out. Second, due to data constraints, we could not conduct a formal mediation analysis to identify the specific pathways through which SHCII reduces vulnerability. Future research using longitudinal panel data and more detailed expenditure information is needed to address these limitations.

## Conclusions

This study provides robust evidence that China’s SHCII effectively reduces vulnerability to medical impoverishment for its direct beneficiaries, with the protective effect concentrated among low-income, rural, older populations, and those with good access to care. No statistically significant effect was observed for urban residents, suggesting that the policy’s marginal benefit may be limited in settings with already higher baseline financial protection. The SHCII implementation model in Shandong is broadly consistent with national guidelines. Therefore, our findings are likely generalizable to provinces with similar policy parameters and rural healthcare infrastructure. Because the protective effect is observed only among patients who exceeded the deductible, provinces with lower deductibles may see a larger share of beneficiaries and potentially stronger population‑level effects, whereas provinces with higher deductibles might observe weaker effects. Similarly, provinces with more generous co‑payment rates could experience greater risk reduction. Regarding rural healthcare infrastructure, the significant effect for rural residents in Shandong suggests that SHCII can be effective in rural settings, but the degree of effectiveness may depend on local primary care capacity and service accessibility. Future research using multi‑province data with varying policy designs and infrastructure levels is needed to test these possibilities.

## Data Availability

The data that support the findings of this study are available from medical management service center of Shandong health commission but restrictions apply to the availability of these data, which were used under license for the current study, and so are not publicly available. Data are however available from the authors upon reasonable request and with permission of medical management service center of Shandong health commission.

## Abbreviations

DID: Difference-in-difference
OOP: Out of-pocket
HDHPs: High-deductible health plans
VHI: Voluntary health insurance
NHS: National Health Service
SHI: Social Health Insurance
RMI: Residents Basic Medical Insurance
BMI: Basic Medical Social Health Insurance
VEP: Vulnerability as Expected Poverty
CHSS: China’s Health Services Survey
